# The Visible Stomach: Elusive Diffuse-Type Adenocarcinoma Presents With Gastric Outlet Obstruction

**DOI:** 10.7759/cureus.25554

**Published:** 2022-05-31

**Authors:** Mhd F Safadi, Hadeel Shamma, Matthias Berger

**Affiliations:** 1 Department of General, Visceral, and Proctological Surgery, DIAKOMED Diakoniekrankenhaus Chemnitzer Land, Hartmannsdorf, DEU; 2 Department of Pathology, Klinikum Chemnitz, Chemnitz, DEU

**Keywords:** carcinoma of the stomach, positron emission tomography/computed tomography, signet-ring cell adenocarcinoma, malignant gastric outlet obstruction, acute massive gastric dilatation, upper endoscopy, adenocarcinoma of the stomach, diagnostic delay, locally advanced gastric cancer, diffuse type

## Abstract

The diagnosis of diffuse-type gastric cancers may be challenging due to their submucosal infiltration. A male in his early 60s was diagnosed with signet-ring cell adenocarcinoma of the diffuse type based on a biopsy from a perforated gastric ulcer. Postoperative workup was negative, including repeated esophagogastroduodenoscopy, gastric biopsies, tumor markers, computed tomography (CT), and positron emission tomography (PET). Six months after the operation, the patient presented to our center with abdominal discomfort and nausea. The clinical examination showed an enlarged visible stomach due to gastric outlet obstruction. The patient underwent total gastrectomy after confirmation of malignancy using an intraoperative frozen section. However, the tumor was already advanced locally and regionally. Confirmed malignancy in biopsies from perforated gastric ulcers should be never considered false positivity. To avoid missing a diffuse gastric cancer, endoscopic biopsies should be obtained using advanced techniques such as submucosal dissection under endosonographic guidance.

## Introduction

The manifestations of gastric cancer include epigastric discomfort, anorexia, weight loss, gastrointestinal bleeding, and gastric outlet obstruction in advanced cases, among others [1]. Peptic ulcer perforation may be the presenting event in 6%-14% of cases [2]. The diffuse type according to Lauren presents about 30% of gastric carcinomas, and it shows aggressive behavior compared to the intestinal type [3].

The diagnosis of gastric cancer is based on histological examination. However, the diffuse type may elude diagnosis because the mucosa may appear endoscopically normal given the submucosal infiltration [3]. This can delay the diagnosis for several months and is usually associated with disease progression and poor prognosis [4].

In this article, we report a patient who was diagnosed with gastric cancer based on a biopsy from a perforated ulcer. The negative postoperative study resulted in a considerable delay in the diagnosis and treatment. In the discussion, we present important pitfalls in approaching the diagnosis of gastric adenocarcinoma.

## Case presentation

The history of the patient dates back to about six months before his presentation to our center. A male in his early 60s with controlled arterial hypertension and diabetes mellitus presented to a local hospital complaining of acute-onset abdominal pain with a rigid abdomen. Based on the clinical and radiological findings, the patient was diagnosed with perforated viscus. The emergency laparotomy showed a perforated prepyloric ulcer with diffuse peritonitis. The ulcer was excised with primary repair, and the patient had an uneventful recovery. The histology showed signet-ring cell adenocarcinoma of the diffuse type according to Lauren’s classification. The diagnosis was confirmed using immunohistochemistry with a positive reaction for pan-cytokeratin and cytokeratin 7 in the muscular layer.

A staging study was initiated based on these results. In the upper gastrointestinal endoscopy up to the descending part of the duodenum, the entire gastric mucosa was intact, and no lesions could be found. The multiple gastric biopsies showed no evidence of malignancy. Similarly, there were no obvious abnormalities in the computed tomography (CT) of the abdomen. Using positron emission tomography with 2-deoxy-2-[fluorine-18]-fluoro-D-glucose integrated with computed tomography (FDG-PET/CT), a whole-body scan was performed from the skull base down to the midthigh. This showed “reactive inflammatory abdominal lymphadenopathy” with otherwise no evidence of malignant activity. A second endoscopy one month later showed no findings, and the biopsies were once again negative. Close clinical follow-up by the general practitioner was arranged. The last endoscopic examination was performed one month before the presentation, and chronic gastritis in the pyloric region was described, again with negative biopsies.

**Figure 1:**
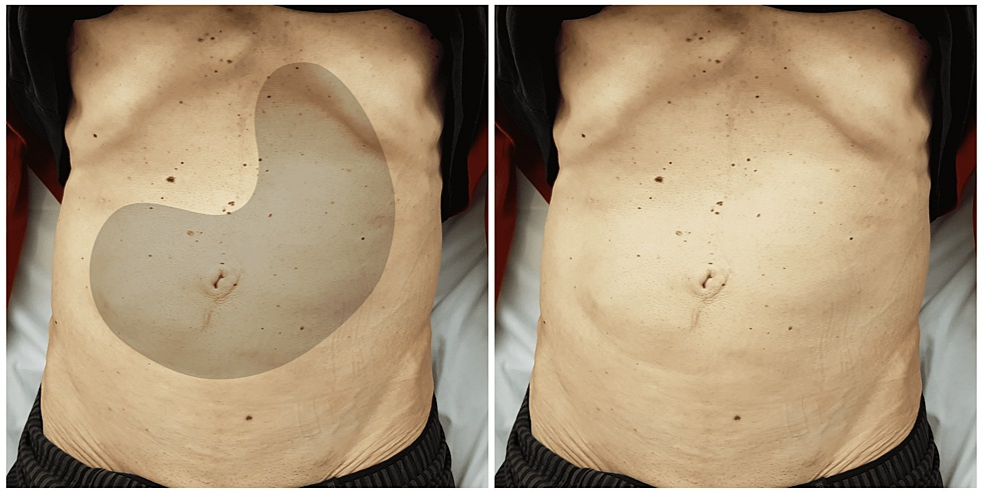
The visible stomach. On the left, the stomach is sketched against the original photo of the abdomen that appears on the right. The enlarged visible stomach occupies the whole upper and middle abdomen and reaches the hypogastric region. An occasional propulsive and peristaltic activity was also visible during the examination. The unremarkable upper abdominal scar can be seen on the midline. Note also the abdominal cachexia with the prominent ribs and costal margin.

The patient presented to the emergency department of our hospital about six months after the initial operation. The presenting complaints included progressive abdominal heaviness and nausea, “which are becoming gradually intolerable” according to the patient. Self-induced vomiting provided some relief. Bowel movements were reported only once every 3-4 days. Further questioning revealed weight loss of about 20 kg in the last six months. Abdominal inspection showed an enlarged visible stomach as a spot diagnosis (Figure 1). A nasogastric tube was passed and produced about 1 L of fluids with undigested food particles.

Investigations

**Figure 2:**
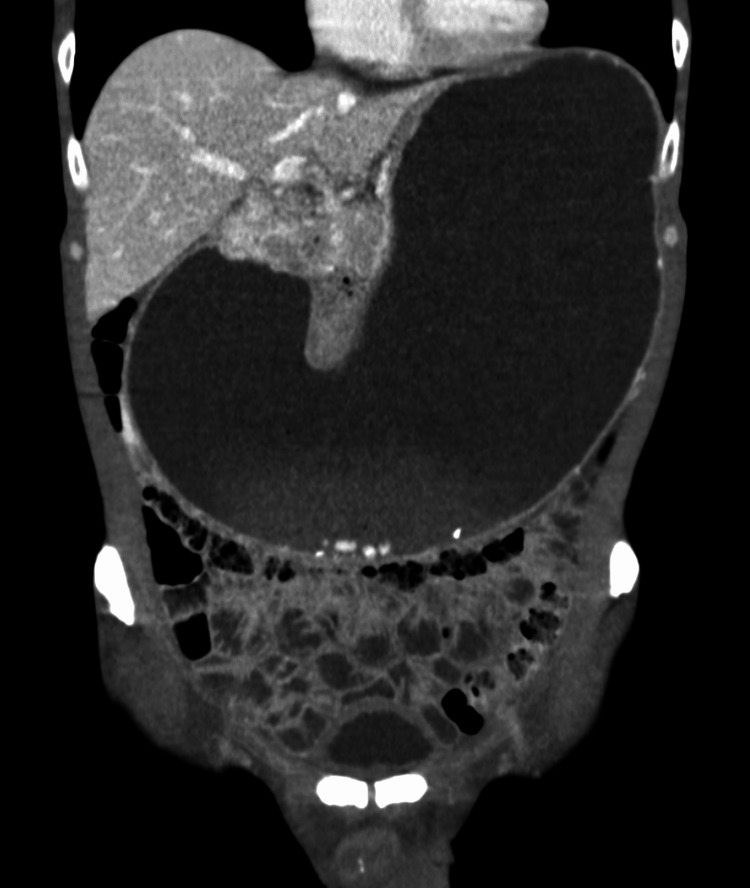
Computed tomography of the abdomen. The figure shows a coronal section of the abdominal computed tomography with intravenous contrast enhancement in the arterial phase. The stomach occupies the upper two-thirds of the abdomen and compromises the abdominal organs, including the intestinal loops in the lower abdomen and the liver in the upper left of the image. Note also the compromised colon throughout its course. A suspected heterogenous mass can be recognized in the pyloric region. The liver is free of metastasis.

All laboratory values, including tumor markers (cancer embryonic antigen (CEA), cancer antigen (CA) 19-9, and CA 72-4), were within the normal limit. The esophagogastroduodenoscopy showed an extensively enlarged stomach. After aspiration of about 2.5 L of turbid fluids, careful inspection of the gastric mucosa up to the prepyloric region showed no abnormalities. Passing the pyloric canal was impossible due to the severe stenosis. In addition to the giant stomach, the computed tomography of the abdomen showed a suspicious mass in the pyloric region (Figure 2).

Differential diagnosis

This patient presented with severe gastric dilatation due to chronic gastric outlet obstruction. From an anatomical point of view, the hindrance can be located anywhere from the pylorus to the duodenum and can result from lumen obstruction, intramural lesions, or extrinsic compression.

Extrinsic lesions could be ruled out in the CT scan: the pancreas was normal, the colon was pushed down, and the other slices of the CT scan excluded a hepatic or biliary mass. Intraluminal lesions (such as gastric polyps and bezoars) could also be ruled out in the gastroscopy up to the pylorus. Therefore, intramural lesions are the most probable cause of the obstruction. These have two main causes in adults: either gastric tumors or benign peptic ulcer disease with pyloric hypertrophy. The differentiation between the two entities based on the CT scan, the endoscopic and histological findings, or the laboratory results was not possible. Given the proven malignancy six months previously, stenosing gastric cancer is the most probable diagnosis. Therefore, an explorative laparotomy was decided for both diagnosis and treatment.

Treatment

**Figure 3:**
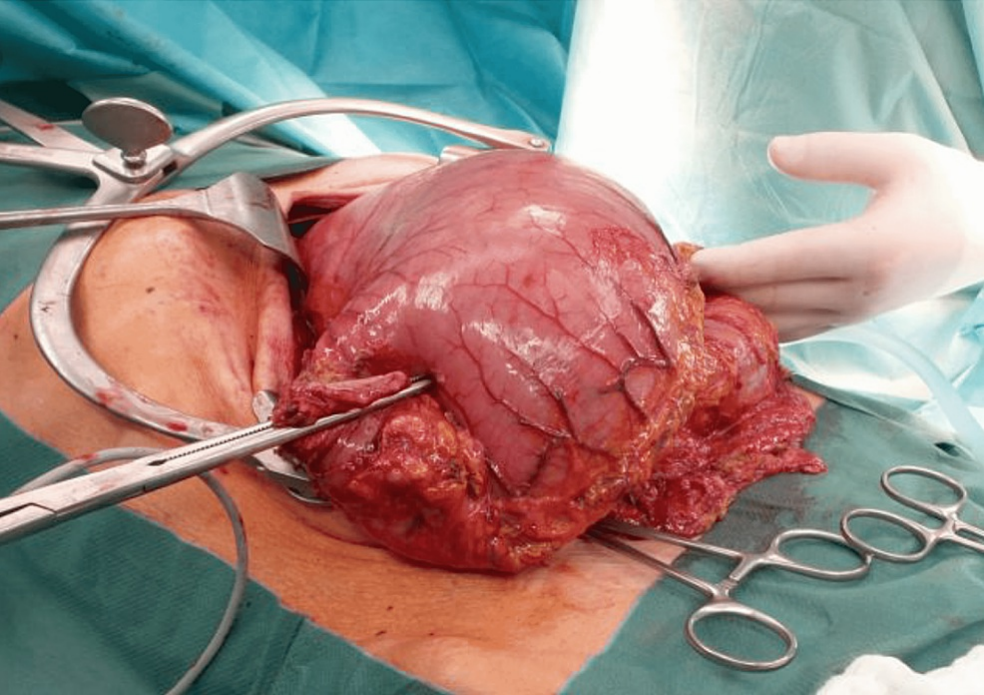
Intraoperative image of the stomach. This intraoperative image shows the huge stomach after the division of the duodenum. After total gastrectomy, the proximal and distal resection margins were free of tumors in the frozen section.

For preoperative optimization, the patient received total parenteral nutrition over a central line with continuous nasogastric decompression for five days. The initial plan included a distal gastrectomy with an intraoperative frozen section (Figure 3). This confirmed the presence of infiltrating adenocarcinoma. Due to the gross lymphadenopathy over the course of the left gastric artery, celiac trunk, and pancreas, we proceeded to total gastrectomy with D2 lymphadenectomy and end-to-side Roux-en-Y oesophagojejunostomy. The postoperative course was unremarkable, and the patient was discharged home on the eighth postoperative day.

The final histological examination showed a poorly differentiated adenocarcinoma of the pylorus with invasion of the perigastric tissue and involvement of 32 out of 37 lymph nodes (pT4b, pN3b, L1, V0, Pn1, stage IV). The patient was referred to adjuvant therapy according to the recommendations of the multidisciplinary tumor board.

## Discussion

Gastric ulcer disease is a common entity that may shield malignancy in less than 1% of cases [5]. Gastric carcinoma is seen in up to 6%-14% of perforated gastric ulcers [2]. In such cases, a perforated ulcer is usually the first manifestation of gastric carcinoma. Generous biopsies from the ulcer’s edges or complete ulcer excision should be performed intraoperatively. Additionally, follow-up gastroscopy in benign ulcers is essential to avoid missed malignancies [5].

According to Lauren’s classification, gastric cancer can be divided into intestinal and diffuse types. The diffuse type accounts for about 30% of cases, and it is characterized by aggressive behavior and poor outcomes [3] since the cells of this type show an enhanced ability to invade the stomach wall and lymphatic vessels [6].

Tissue diagnosis using endoscopic biopsies is the standard diagnostic modality for gastric cancer. This is usually straightforward in the presence of mucosal lesions or ulcers, but it can be challenging in the diffuse type, which spreads submucosally and evades detection [7]. In these cases, upper endoscopy may be nondiagnostic, and mucosal biopsies can be false negative, as in our patient. For this reason, more invasive methods should be used in highly suspected cases. A strip-and-bite biopsy can be taken by resecting the overlying mucosa to expose the submucosal layer, and then, a bite biopsy is obtained from the surface [7]. Submucosal dissection is another technique that is performed by injecting epinephrine, dissecting the submucosa using an endoscopic knife, and obtaining the needed tissue [8]. Such techniques would provide a conclusive diagnosis in up to 81% of patients with diffuse-type cancer [9].

The results of immunohistochemistry play an important role in the histological evaluation of malignant tumors. In the initial biopsy obtained from the perforated ulcer in this patient, the muscular layer showed a positive reaction to pan-cytokeratin and cytokeratin 7. Although these markers are not specific to gastric cancer and can also be reactive in other epithelial neoplasms, their presence confirms malignancy and leaves no room for assumptions [10]. Therefore, these results should not have been ignored, and more rigorous investigations should have been carried out.

Endosonography is another important investigation that was unfortunately not performed in the workup of this patient. In their study of 32 patients, Will et al. reported an accuracy of 87.5% in detecting diffuse gastric cancers compared to only 58% when using endoscopic biopsies alone [11]. The reported changes include loss of layer contour, minor destruction of the layer’s architecture, and enlargement with the folding of the submucosal and muscular layers [11]. Additionally, endosonographic guidance can facilitate obtaining deep biopsies from the suspected areas and improve the diagnostic yield [9].

Serum tumor markers can be used for follow-up after the treatment of gastric cancer, but they have no role in the preoperative diagnosis or staging because of their low sensitivity and specificity [12]. For example, cancer embryonic antigen (CEA) can be elevated in only 14%-25% of gastric cancers [12,13] and in up to 35.7% of advanced tumors [14]. Cancer antigen 72-4 (CA 72-4) was positive in 2.3% of early cancers and in 37.5% of advanced cancers. Even with combining multiple markers, only about half of the patients will show abnormal levels [14]. Therefore, tumor markers should not be used as a tool for excluding gastric cancer, and normal levels do not rule out malignancy.

Positron emission tomography is a useful modality for detecting regional and distant metastasis in gastric cancer, and it can upgrade the staging and change the treatment plan compared with CT scan alone [15]. However, FDG-PET/CT should not be used as a screening tool for gastric cancer; it should only be used to determine the activity of the already confirmed malignancy. Serrano et al. found that the primary tumor was visible on a PET scan in only 72% of 608 patients [16]. Due to the variations in the metabolic activity, not all tumors are FDG-avid. The affinity for FDG is even lower in the diffuse type and in mucinous adenocarcinoma with signet-ring cells [17]. Therefore, and as seen in this case, a PET scan should not be employed as an exclusion tool for a suspected adenocarcinoma, and a negative study does not rule out gastric cancer.

Delayed diagnosis of gastric adenocarcinoma was discussed extensively in the literature. Missing gastric cancer on upper endoscopy is reported in 5%-10% of cases, most commonly with the diffuse type [4]. In their prospective study, Zilling et al. found that the diagnosis was delayed for more than three months in 24% of patients because of ongoing investigations [18]. We believe that earlier recognition of the disease would have been possible in this patient if more aggressive diagnostic measures were taken after the confirmation of malignancy in the initial biopsies. It is important to stress that knowledge or skill gaps by the provider may be responsible for such a delay in about half of the cases [1]. Accordingly, it seems that the lack of knowledge and experience of the initial treating physicians was the main factor that led to the diagnostic delay in our patient.

Because of its lower morbidity, diagnostic laparoscopy is recommended in advanced gastric cancers to exclude peritoneal metastasis, as it may help avoid an unnecessary laparotomy in about one-third of patients [19]. We omitted this step because surgical management of the gastric outlet obstruction was required. The frozen section is a valuable method for intraoperative confirmation of suspected gastric cancer. Since it may be difficult to differentiate poorly-differentiated and signet-ring cell variants from benign inflammatory cells, many morphologic features were described to avoid misdiagnosis [20]. Frozen section examination also allows an intraoperative confirmation of free proximal and distal resection margins before restoring the continuity, although false-negative results were reported in less than 1% of cases [20].

## Conclusions

The diagnosis of gastric cancer can be sometimes delayed, which results in disease progression and poor outcomes. All gastric ulcers found on endoscopy should be adequately biopsied. Full-thickness biopsies should be generously obtained from every perforated gastric ulcer to exclude malignancy. Positive results should be considered a proven malignancy even when the endoscopic biopsies are negative. In suspected cases, advanced sampling techniques should be applied. Endosonography is also a useful adjunct to endoscopy for diagnosing suspected gastric cancer in the absence of mucosal lesions, especially if a previous biopsy demonstrated malignancy. Lastly, serum tumor markers or imaging modalities such as positron emission tomography cannot reliably exclude suspected gastric cancer.
